
JOURNAL INFORMATION
==============================
NLM Title Abbreviation: ScientificWorldJournal
Journal ID: ScientificWorldJournal
NLM Journal ID: 101131163
Journal ID: TSWJ

The Scientific World Journal

ISSN: 2356-6140
EISSN: 1537-744X
Publisher: Wiley

ARTICLE INFORMATION
==============================
PMCID: PMC6083952
PMID: 30147528
DOI: 10.1100/tsw.2001.177
Article ID: 317179
Article version: 1
Subjects: Short Report

Chronic Apoptotic Signaling is Induced by Low Levels of Mitochondrial Dna Mutations in the Mouse Heart

Zhang Dekui
Mott Justin L.
Zassenhaus H. Peter *
Department of Molecular Microbiology and Immunology, St. Louis University, 1402 S. Grand Blvd., St. Louis, MO 63104, Spain
*H. Peter Zassenhaus: zassenp@slu.edu
Publication date: 2001
Electronic publication date: 2001 May 1
Volume: 1
First page: 59
Last page: 59
Copyright: Copyright © 2001 Dekui Zhang et al.
Copyright year: 2001
License: This is an open access article distributed under the Creative Commons Attribution License, which permits unrestricted use, distribution, and reproduction in any medium, provided the original work is properly cited.
License URL: https://creativecommons.org/licenses/by/3.0/

==============================
Miami *Nature Biotechnology* Short Reports
*TheScientificWorld* (2001) 1(S3), 59SR
ISSN 1532-2246; DOI 10.1100/tsw.2001.177

### CHRONIC APOPTOTIC SIGNALING IS INDUCED BY LOW LEVELS OF MITOCHONDRIAL DNA MUTATIONS IN THE MOUSE HEART

Dekui Zhang, Justin L. Mott, and H. Peter Zassenhaus*

Department of Molecular Microbiology and Immunology, St. Louis University, 1402 S. Grand Blvd., St. Louis, MO 63104
* zassenp@slu.edu

**INTRODUCTION.** MtDNA mutations rise with age and are associated with a variety of degenerative diseases. Their pathogenesis has been questioned, however, in part because it is not clear by what mechanism low levels of mtDNA mutations cause cellular dysfunction. In order to study this issue, we constructed transgenic (tg) mice that rapidly accumulate both point and deletion mutations in cardiac mtDNA starting at birth due to the expression of a proofreading deficient DNA pol $\gamma$ (1). By 1 month of age, mutations are at levels commonly seen in the aged or ischemic human heart (2). Tg mice develop dilated cardiomyopathy but pathogenesis does not appear to involve either mitochondrial respiratory dysfunction or increased oxidative stress (3). Rather, the rising frequency of mtDNA mutations induces chronic pro-apoptotic signaling in all cardiomyocytes, some of which succumb by apoptosis. This mitochondrial signaling is counteracted by a persistent upregulation of a number of anti-apoptotic proteins. Although myocytic apoptosis is thereby blunted, we propose that the "compensated state" contributes to myocytic dysfunction.

**METHODS.** Protein levels were quantified by western blotting of heart lysates or sub-cellular fractions. Cytochrome c (*cyt c*) release from mitochondria was characterized by gel filtration of S100 fractions. The proportion of cardiomyocytes showing elevated pro- and anti-apoptotic signaling was analyzed by immunohistochemistry and TUNEL staining.

**RESULTS.** Apoptosis in the transgenic heart begins to rise relative to controls at 3 weeks of age, peaks 1 week later, and then declines at a time when Bcl2 levels rise and remain high (Fig. 1). Apoptosis is associated with the release of *cyt c* and translocation of BAX onto mitochondria (Fig. 2). Counteracting these pro-apoptotic signals is the upregulation of a number of anti-apoptotic proteins which inhibit different steps in mitochondrial apoptotic signaling (Fig. 2): Bfl1 (BAX activity {4}), Bcl2 and Bcl-xL (BAX-induced release of *cyt c* {5}), Hsp27 (activation of caspase 9 {6}), XIAP (activation of caspase 3 {7}). Although total levels of both Bcl2 and Bcl-xL are elevated, only Bcl2 is also upregulated in mitochondria (Fig. 2). Immunohistochemical analysis for Bcl2 and *cyt c* shows that by 5 weeks of age all cardiomyocytes in the transgenic heart show upregulation of Bcl2 and release of *cyt c*.

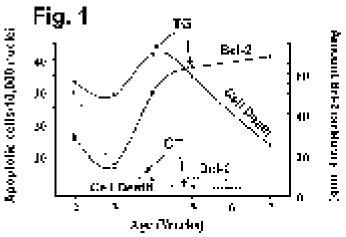

**Fig. 1: Mitochondrial DNA mutations cause apoptosis and an anti-apoptotic response.** Apoptotic cell death increases in transgenic (TG) versus control (CT) mice when TG mice average one point mutation per mitochondrial genome. The protective protein Bcl-2 is then upregulated, and cell death begins to subside. Still, apoptosis remains high in TG mice to at least 11 weeks-of-age.

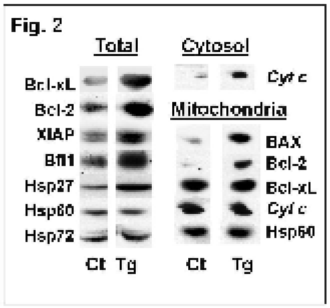

**Fig. 2: Apoptosis-regulating proteins achieve a new balance in mice with mtDNA mutations.** Proteins known to protect against apoptosis (Bcl-xL, Bcl-2, XIAP, Bfl-1, and HSP27) are upregulated at the whole heart (total) level. HSPs 60 and 72 show no change, demonstrating that changes reflect a specific response. Despite this heightened protection, we see increased release of cytochrome c (cytosol) and increased proapoptotic BAX on mitochondria. Much of the Bcl-2 increase is due to mitochondrially localized protein, while the Bcl-xL response is not targeted to mitochondria. Specificity controls in the mitochondrial fraction are *cyt c* and HSP 60.

**SUMMARY.** Rising levels of mtDNA mutations cause chronic apoptosis in the mouse heart, possibly mediated by the release of *cyt c*. This pro-apoptotic signal is counteracted by a persistent upregulation of anti-apoptotic proteins that inhibit different steps in the apoptotic pathway. This altered balance may establish a compensated state that contributes to the pathogenesis of mtDNA mutations. Supported by grants from the American Heart Association, the Alzheimer Association, and the Aging and Heart, Lung & Blood Institutes of the NIH.

(Additional figures at end of paper.)

**REFERENCES**.

1. Mott, J.L. et al. (1999) Ann. N. Y. Acad. Sci. 893, 353-357
2. Zhang, D. et al. (2000) Genomics 69, 151-161
3. Mott, J.L. et al. Mutation Res. (in press)

4. Holmgreen, S.P. et al. (1999) Cell Death Differ. 6, 525-532
5. Gross, A. et al. (1999) Genes Dev. 13, 1899-1911
6. Garrido, C. et al. (1999) FASEB J. 13, 2061-2070
7. Deveraux, Q.L. et al. (1999) J. Clin. Immunol. 19, 388-398

Fig. 3

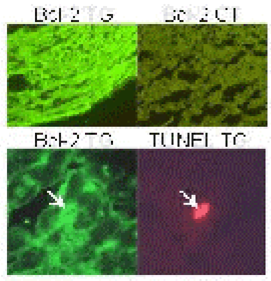

**Fig. 3: Mitochondrial DNA mutations lead to cytochrome c release in all cardiomyocytes.** Cytochrome c release was monitored as an increase in *cyt c* immunoreactivity in paraffin-embedded slides. Note the overall increase in cyt c immunoreactivity in the transgenic heart (TG) at 5 weeks-of-age compared to the control heart (CT). As the amount of *cyt c* in transgenic hearts – either in mitochondria or in whole cell extracts – is at most only slightly higher than controls, we interpret the increased immunoreactivity as an indicator of *cyt c* release. Importantly, at 3 weeks of age, a patchy cellular pattern of increased immunofluorescence is observed, as would be expected if the rising frequencies of mtDNA mutations need to cross a threshold to induce *cyt c* release. Given the stochastic mechanism causing mtDNA mutations in this model, not all cells are expected to arrive at that threshold at the same age. By 5 weeks of age, all cells appear to be involved in the disease process. Given the cytotoxicity of released *cyt c*, cell death remains remarkably in check due to a protective response.

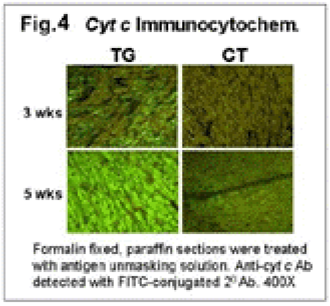

**Fig. 4: All myocytes receive protection from apoptosis.** The anti-apoptotic protein Bcl-2 is upregulated in all myocytes in transgenic mice with mtDNA mutations (TG), as seen by immunofluorescence. In fact, even dying cells (TUNEL positive; red) show increased Bcl-2 expression, and therefore do not have a relative deficiency of Bcl-2-like protection.
